# A New Extended-*X* Family of Distributions: Properties and Applications

**DOI:** 10.1155/2020/4650520

**Published:** 2020-05-26

**Authors:** Mi Zichuan, Saddam Hussain, Anum Iftikhar, Muhammad Ilyas, Zubair Ahmad, Dost Muhammad Khan, Sadaf Manzoor

**Affiliations:** ^1^School of Statistics, Shanxi University of Finance and Economic, Taiyuan, China; ^2^Department of Statistics, University of Malakand, Dir (L), KP, Pakistan; ^3^Department of Statistics, Yazd University, P.O. Box 89175-741, Yazd, Iran; ^4^Department of Statistics, Abdul Wali University Mardan, KP, Pakistan; ^5^Department of Statistics, Islamia College Peshawar, KP, Pakistan

## Abstract

During the past couple of years, statistical distributions have been widely used in applied areas such as reliability engineering, medical, and financial sciences. In this context, we come across a diverse range of statistical distributions for modeling heavy tailed data sets. Well-known distributions are log-normal, log-*t*, various versions of Pareto, log-logistic, Weibull, gamma, exponential, Rayleigh and its variants, and generalized beta of the second kind distributions, among others. In this paper, we try to supplement the distribution theory literature by incorporating a new model, called a new extended Weibull distribution. The proposed distribution is very flexible and exhibits desirable properties. Maximum likelihood estimators of the model parameters are obtained, and a Monte Carlo simulation study is conducted to assess the behavior of these estimators. Finally, we provide a comparative study of the newly proposed and some other existing methods via analyzing three real data sets from different disciplines such as reliability engineering, medical, and financial sciences. It has been observed that the proposed method outclasses well-known distributions on the basis of model selection criteria.

## 1. Introduction

In the practice of statistical theory, particularly, in engineering, medical, and financial sciences, data modeling is an interesting research topic. In this context, the statistical distributions are worthwhile for modeling such data sets. The most frequently used statistical distributions are exponential, Rayleigh, Weibull, beta, gamma, log-normal, Pareto, Lomax, and Burr, among others. However, these traditional distributions are not flexible enough for countering complex forms of the data sets. For example, in reliability engineering and biomedical sciences, the data sets are usually unimodal and skewed to the right; see Demicheli et al.'s [1], Lai and Xie's [2], Zajicek's [3], and Almalki and Yuan's [4] studies. Hence, in such cases, the utilization of the exponential, Rayleigh, Weibull, or Lomax distributions may not be a suitable choice to employ. On the other hand, the gamma, beta, and log-normal distributions do not have closed forms for the cumulative distribution function (cdf) causing difficulties in estimating the parameters.

Furthermore, in financial and actuarial risk management problems, the data sets are usually unimodal, skewed to the right, and possess thick right tail; for details see, Cooray and Ananda's [5] and Eling's [6] studies, among others. The distributions that exhibit such characteristics can be used quite effectively to model insurance loss data to estimate the business risk level. The distributions commonly used in the literature include Pareto by Cooray and Ananda [5], Lomax by Scollnik [7], Burr by Nadarajah and Bakar [8], and Weibull by Bakar et al. [9], which are particularly appropriate for modeling of insurance losses, financial returns, file sizes on the network servers, etc. Unfortunately, these distributions are subject to some sort of deficiencies. For example, the Pareto distribution, due to the monotonically decreasing shape of the density, does not provide the best fit in many applications, whereas the Weibull model is capable of covering the behavior of small losses, but fails to cover the behavior of large losses.

Moreover, Dutta and Perry [10] provided an empirical study on loss distributions using exploratory data analysis and other empirical approaches to estimate the risk. They rejected the idea of using exponential, gamma, and Weibull distributions due to their poor results and pointed out that one would need to use a model that is flexible enough in its structure. Hence, there are only few probability distributions capable of modeling heavy tailed data sets and none of them are flexible enough to provide greater accuracy in fitting complex forms of data.

To address the problems stated above, the researchers have shown an increased interest in defining new families of distributions by incorporating one or more additional parameters to the well-known distributions. The new families have been defined through many different approaches introducing additional, location, scale, shape, and transmuted parameters, to generalize the existing distributions. These generalizations are mainly based on, but not limited to, the following approaches: (i) transformation of the variable and (ii) compounding of two or more models; in detail, we refer the interested readers to studies by Tahir and Cordeiro [11], Bhati and Ravi [12], and Ahmad et al. [13].

One of the most interesting methods of adding the shape parameter to the existing distributions is exponentiation. The exponentiated family pioneer to Mudholkar and Srivastava [14] is defined by the following cdf:

(1) $$\begin{array}{c} G\left(x;a,\xi \right)=F{\left(x;\xi \right)}^{a},\;a,\xi >0,x\in R, \end{array}$$

where *a* is the additional shape parameter.

Marshall and Olkin [15] pioneered a new simple approach of introducing a single-scale parameter to a family of distributions. The cdf of the Marshall-Olkin (MO) family is given by

(2) $$\begin{array}{c} G\left(x;\sigma ,\xi \right)=\frac{F\left(x:\xi \right)}{\sigma +\left(1-\sigma \right)F\left(x;\xi \right)},\;\sigma ,\xi >0,x\in R, \end{array}$$

where *σ* is the additional scale parameter.

Cordeiro and Castro proposed (2010) proposed the Kumaraswamy-*G* family defined by

(3) $$\begin{array}{c} G\left(x;a,b,\xi \right)=1-{\left(1-F{\left(x;\xi \right)}^{a}\right)}^{b},\;a,b,\xi >0,x\in R, \end{array}$$

where *a* and *b* are the additional shape parameters.

Mostly, so far in the literature either the scale or shape parameters are introduced to propose a new family of distributions. Introducing both the scale and shape parameters to a family of distribution may increase the level of flexibility. But the number of parameters increases, and the estimation of parameters and computation of many mathematical properties become complicated.

In the premises of above, a new attempt has been made to introduce more flexible probability distributions by introducing a single additional parameter which serves as a scale as well as a shape parameter and provides greater accuracy in fitting real-life data in applied fields such as reliability engineering, medical, and financial sciences. Hence, in this paper, a new method is proposed to introduce new statistical distributions. The proposed family may be named as a new extended-*X* (NE-*X*) family. A random variable *X* is said to follow the proposed family, if its cdf is given by

(4) $$\begin{array}{c} G\left(x;\theta ,\xi \right)=1-{\left\{\frac{1-F{\left(x;\xi \right)}^{2}}{1-\left(1-\theta \right)F{\left(x;\xi \right)}^{2}}\right\}}^{\theta },\;\theta >0,x\in R. \end{array}$$

The introduction of the additional parameter *θ* in expression (4) adds greater distributional flexibility to the baseline distributions with cdf *F*(*x*; *ξ*) which may depend on the vector parameter *ξ*. The additional parameter plays the role of both scale and shape parameters. The probability density function (pdf) corresponding to (4) is

(5) $$\begin{array}{c} g\left(x,\theta ,\xi \right)=\frac{2\theta^{2}f\left(x;\xi \right)F\left(x;\xi \right){\left\{1-F{\left(x;\xi \right)}^{2}\right\}}^{\theta -1}}{{\left\{1-\left(1-\theta \right)F{\left(x;\xi \right)}^{2}\right\}}^{0+1}},\;x\in R. \end{array}$$

We concentrate our focus to a special submodel of the proposed family, called a new extended Weibull (NE-W) distribution.

Finally, we direct our attention to the results related to the NE-W model with real life data in three different disciplines. The first data set is taken from biomedical field, and the results of the proposed model are compared with five other competitive models including (i) two-parameter Weibull distribution and (ii) three-parameter models such as flexible Weibull extended (FWE), alpha power transformed Weibull (APTW), Marshall-Olkin Weibull (MOW), and modified Weibull (MW) distributions. The second data set is taken from reliability engineering, and the results of the proposed model are compared with three other well-known distributions such as (i) the three-parameter extended alpha power transformed Weibull (Ex-APTW), (ii) four-parameter Kumaraswamy Weibull (Ku-W), and (iii) beta Weibull (BW) distributions. The third data set is taken from financial sciences, and the results of the proposed model are compared with the Weibull and other heavy tailed models including Lomax and Burr-XII (B-XII) distributions.

The rest of the paper is organized as follows: in Section 2, a special case of the proposed family is introduced and the shapes of its density and hazard functions are investigated. Some mathematical properties of the proposed family are derived in Section 3. Maximum likelihood estimators of the model parameters are obtained in Section 4. In the same section, a Monte Carlo simulation study is conducted. Practical applications are analyzed in Section 5. Here, the NE-W distribution is compared with the models mentioned above under different measures of discrimination and other goodness of fit measures. Finally, some concluding remarks are given in the last section.

## 2. Model Description

In this section, we introduce the NE-W distribution. Considering the cdf of the two-parameter Weibull distribution with the shape parameter *α* > 0 and scale parameter *γ* > 0, given by *F*(*x*; *ξ*) = 1 − *e*^−*γx*^*a*^^, *x* ≥ 0, and pdf, given by *f*(*x*; *ξ*) = *aγx*^*a*−1^ *e*^−*γx*^*a*^^, respectively, where *ξ* = (*α*, *γ*). Then, the cdf of the NE-W distribution is given by

(6) $$\begin{array}{c} G\left(x;\theta ,\xi \right)=1-{\left(\frac{1-{\left(1-e^{-\gamma x^{a}}\right)}^{2}}{1-\left(1-\theta \right){\left(1-e^{-\gamma x^{a}}\right)}^{2}}\right)}^{\theta },\;x\geq 0,a,\theta ,\gamma >0. \end{array}$$

The density function of the NE-W distribution is

(7) $$\begin{array}{c} g\left(x;\theta ,\xi \right)=\frac{2\theta^{2}a\gamma x^{a-1}e^{-\gamma x^{a}}\left(1-e^{-\gamma x^{a}}\right){\left\{1-{\left(1-e^{-\gamma x^{a}}\right)}^{2}\right\}}^{\theta -1}}{{\left\{1-\left(1-\theta \right){\left(1-e^{-\gamma x^{a}}\right)}^{2}\right\}}^{\theta +1}},\;x>0. \end{array}$$

**Figure 1:**
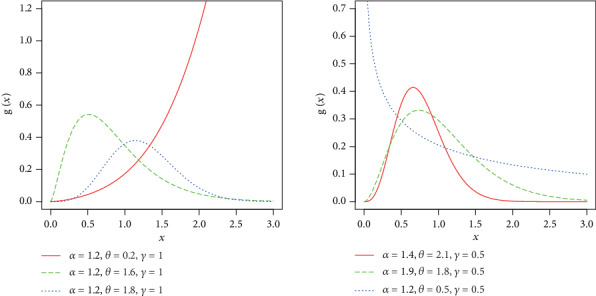
Some possible shapes of the density function of the NE-W distribution.

**Figure 2:**
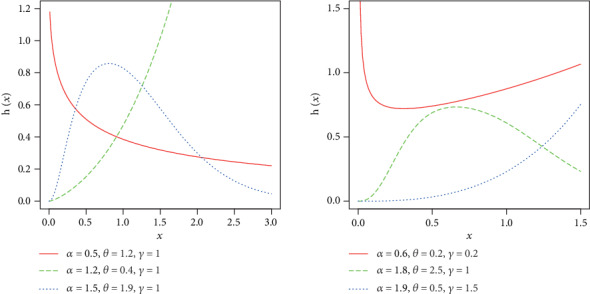
Some possible shapes for the hazard function of the NE-W distribution.

Some possible shapes for the density and hazard functions of the NE-W distribution are sketched in Figures 1 and 2, respectively,

In Figure 1, we plotted different shapes for the density of NE-W distribution. When *α*, *θ* < 1, then the density of the proposed model behaves like exponential distribution. But as the value of these parameters increases, the proposed model captures the characteristics of the Rayleigh and Weibull distributions. However, the proposed model has certain advantages over these distributions, since it provides the best fit to data in different disciplines as shown in Section 5. The hrf is plotted in Figure 2. The hazard function of the proposed model is very flexible in accommodating different shapes, namely, decreasing, increasing, unimodal, and bathtub; hence, the NE-W distribution becomes an important model to fit several real lifetime data in applied areas such as reliability, survival analysis, economics, and finance.

## 3. Mathematical Properties of the NE-X Distributions

In this section, we study some mathematical properties of the NE-X distributions such as the quantile function, *r*^th^ moment, and moment generating function.

### 3.1. Quantile Function

The quantile function of the NE-X distributions is given by

(8) $$\begin{array}{c} x_{q}=Q\left(u\right)=G^{-1}\left(u\right)=F^{-1}{\left\{\frac{{\left(1-u\right)}^{1/\theta }-1}{\left(1-\theta \right){\left(1-u\right)}^{1/\theta }-1}\right\}}^{1/2}, \end{array}$$

where *u* ∈ (0, 1). From expression (8), we can see that the proposed model has a closed form solution of the quantile function which makes it easier to generate random numbers for the subcase of the NE-X family.

### 3.2. Moments

This subsection deals with the derivation of *r*^th^ moment of the NE-X distributions. The *r*^th^ moment of the NE-X distributions is derived as

(9) $$\begin{array}{c} \mu_{r}^{/}=\int_{-\infty }^{\infty }x^{r}g\left(x;\theta ,\xi \right)\;dx. \end{array}$$

Using (5) in (9), we have

(10) $$\begin{array}{c} \mu_{r}^{/}=\int_{-\infty }^{\infty }x^{r}\frac{2\theta^{2}f\left(x;\xi \right)F\left(x;\xi \right){\left\{1-F{\left(x;\xi \right)}^{2}\right\}}^{\theta -1}}{{\left\{1-\left(1-\theta \right)F{\left(x;\xi \right)}^{2}\right\}}^{0+1}}dx. \end{array}$$

Using the expansion (https://math.stackexchange.com/questions/1624974/series-expansion-1-1-xn)

(11) $$\begin{array}{c} \frac{1}{{\left(1-x\right)}^{n}}=\overset{\infty }{\underset{i=0}{\sum }}\left(\begin{array}{c} i+n-1\\ n-1 \end{array}\right)x^{i} \end{array}$$

and using *x* = (1 − *θ*)*F*(*x*; *ξ*)^2^ and *n* = *θ* + 1 in (11), we get

(12) $$\begin{array}{c} \frac{1}{{\left(1-\left(1-\theta \right)F\left(x;\xi \right)\right)}^{\theta +1}}=\overset{\infty }{\underset{i=0}{\sum }}\left(\begin{array}{c} i+\theta \\ \theta \end{array}\right){\left(1-\theta \right)}^{i}F{\left(x;\xi \right)}^{2i}. \end{array}$$

Also using the series representation

(13) $$\begin{array}{c} {\left(1-y\right)}^{m}=\overset{m}{\underset{j=0}{\sum }}{\left(-1\right)}^{j}\left(\begin{array}{c} m\\ j \end{array}\right)x^{j} \end{array}$$

and using *y* = *F* (*x*; *ξ*)^2^ and *m* = *θ* − 1 in (13), we get

(14) $$\begin{array}{c} {\left(1-F{\left(x;\xi \right)}^{2}\right)}^{\theta -1}=\overset{\theta -1}{\underset{j=0}{\sum }}\left(\begin{array}{c} 0-1\\ j \end{array}\right){\left(-1\right)}^{j}F{\left(x;\xi \right)}^{2j}. \end{array}$$

Using (12) and (14) in (10), we have

(15) $$\begin{array}{c} \mu_{r}^{/}=2\theta^{2}\overset{\infty }{\underset{i=0}{\sum }}\overset{\theta -1}{\underset{j=0}{\sum }}\left(\begin{array}{c} 0-1\\ j \end{array}\right)\left(\begin{array}{c} i+0\\ 0 \end{array}\right){\left(-1\right)}^{j}{\left(1-\theta \right)}^{i}K_{r,2\left(i+j\right)+1}, \end{array}$$

where

(16) $$\begin{array}{c} K_{r,2\left(i+j\right)+1}=\int_{-\infty }^{\infty }x^{r}f\left(x;\xi \right)F{\left(x;\xi \right)}^{2\left(i+j\right)+1}dx. \end{array}$$

Numerical values for the mean, variance, skewness (Sk), and kurtosis (Kur) of the NE-W distribution for some selected values of the parameters are given in Tables 1 and 2. To check the effect of the additional parameter on Sk and Kur, (i) we kept the parameters *α* and *γ* constant and allow *θ* to vary and then (ii) we kept constant the parameters *θ* and *γ* and allow *α* to vary.

From the numerical results provided in Table 1, it is clear that as the additional parameter *θ* increases the mean and variance decrease, whereas increasing *θ* results in increasing the Sk and Kur of the model showing that the proposed distribution is leptokurtic, unimodal, and skewed to the right. From the results provided in Table 1, we can also detect that increase in the parameter *θ* results in producing skewness to the right indicating heavy tail to the right. Also, from the results in Table 2, we can see that as the parameter *α* increases, the distribution produces skewness to the right but has low impact on skewness and kurtosis. Hence, from the numerical results presented in Tables 1 and 2, we conclude that the introduction of the additional parameter to the Weibull model brings more flexibility to the skewness and kurtosis of the NE-W distribution.

The moment generating function, say *M*_*X*_ (*t*), of the NE-W distributions can be obtained as follows:

(17) $$\begin{array}{c} M_{X}\left(t\right)=\overset{\infty }{\underset{r=0}{\sum }}\frac{t^{r}}{r!}\mu_{r}^{/}. \end{array}$$

Using (15) in (17), we get the mgf of the NE-W distributions.

## 4. Maximum Likelihood Estimation and Simulation Study

This section offers the maximum likelihood estimators of the model parameters and provide Monte Carlo simulation study to assess the behavior of these estimators.

### 4.1. Maximum Likelihood Estimation

Numerous approaches for estimating the unknown parameters have been proposed in the literature. Among them, the maximum likelihood estimation is the most prominent and commonly employed method to obtain the point estimators. The maximum likelihood estimators (MLEs) possess desirable properties and can be utilized for constructing the confidence interval and other statistical tests. By MLEs, various statistics are built for assessing the goodness-of-fit in a model, such as the maximum log-likelihood (${\hat{\ell }}_{\max}$), Akaike Information Criterion (AIC), and Bayesian Information Criterion (BIC), as given in the next section. The normal approximation of the MLEs can easily be treated either numerically or analytically. In this subsection, we consider the estimation of the unknown parameters of the NE-X family from complete samples only by the method of maximum likelihood. Suppose *x*_1_, *x*_2_, ⋯, *x*_*n*_ form an observed random sample from the NE-X family with pdf (5). Let Θ = (*α*, *γ*, *θ*)^*T*^ be the 3 × 1 parameter vector. The log likelihood function corresponding to (5) is given by

(18) $$\begin{array}{c} l_{n}\left(\Theta \right)=n\log\left(2\right)+2n\log\theta +\overset{n}{\underset{i=1}{\sum }}\log f\left(x_{i};\xi \right)+\left(0-1\right)\overset{n}{\underset{i=1}{\sum }}\log\left\{1-F{\left(x_{i};\xi \right)}^{2}\right\}+\overset{n}{\underset{i=1}{\sum }}\log F\left(x;\xi \right)-\left(0+1\right)\overset{n}{\underset{i=1}{\sum }}\log\left\{1-\left(1-\theta \right)F{\left(x;\xi \right)}^{2}\right\}. \end{array}$$

The log-likelihood function can be maximized directly either by using the ASS (PROC UNMIXED) or by solving the nonlinear likelihood equations obtained by differentiating (18). The partial derivatives of (18) are as follows:

(19) $$\begin{array}{c} \frac{\partial \ell_{n}\left(\Theta \right)}{\partial \theta }=\frac{2n}{\theta }+\overset{n}{\underset{i=1}{\sum }}\log\left\{1-F{\left(x_{i};\xi \right)}^{2}\right\}-\overset{n}{\underset{i=1}{\sum }}\log\left\{1-\left(1-\theta \right)F{\left(x_{i};\xi \right)}^{2}\right\}-\left(\theta +1\right)\overset{n}{\underset{i=1}{\sum }}\frac{F{\left(x_{i};\xi \right)}^{2}}{\left\{1-\left(1-\theta \right)F{\left(x_{i};\xi \right)}^{2}\right\}},\\ \frac{\partial \ell_{n}\left(\Theta \right)}{\partial \theta }=\overset{n}{\underset{i=1}{\sum }}\frac{\left(\partial f\left(x_{i};\xi \right)\right)/\partial \xi }{\partial f\left(x_{i};\xi \right)}-\left(\theta -1\right)\overset{n}{\underset{i=1}{\sum }}\frac{\left(\partial F{\left(x_{i};\xi \right)}^{2}\right)/\partial \xi }{\left\{1-F{\left(x_{i};\xi \right)}^{2}\right\}}+\overset{n}{\underset{i=1}{\sum }}\frac{\left(\partial F\left(x_{i};\xi \right)\right)/\partial \xi }{\partial F\left(x_{i};\xi \right)}+\left(0+1\right)\overset{n}{\underset{i=1}{\sum }}\frac{\left(\left(1-\theta \right)\partial F{\left(x_{i};\xi \right)}^{2}\right)/\partial \xi }{\left\{1-\left(1-\theta \right)F{\left(x_{i};\xi \right)}^{2}\right\}}. \end{array}$$

Equating the nonlinear system of equations (*∂ℓ*_*n*_(Θ))/*∂θ* and (*∂ℓ*_*n*_(Θ))/*∂ξ* to zero and solving these expressions simultaneously yield the MLEs $\hat{\theta }$ and $\hat{\xi }$, respectively. From expressions (19), it is clear that these expressions are not in explicit forms. Therefore, computer software can be used to solve these expressions numerically. We use optim() R-function with the argument method = ^"^SANN^"^ to obtain the maximum likelihood estimators. The expression (18) can be used to obtain the MLEs for any subcase of the proposed family. For the NE-W distribution, the expressions for the MLEs are derived in the appendix.

### 4.2. Monte Carlo Simulation Study

In this subsection, we investigate the performance of the maximum likelihood estimators of the proposed distribution. For the simulation purposes, the NE-W distribution is considered. We use the inverse cdf method for generating random numbers from the NE-W distribution. If *U* ~ *U*(0, 1) and if *G* has an inverse function, then

(20) $$\begin{array}{c} x={\left(\frac{-\log\left[1-{\left(\left(1-u^{1/\theta }-1\right)/\left(\left(1-\theta \right){\left(1-u\right)}^{1/\theta }-1\right)\right)}^{1/2}\right]}{\gamma }\right)}^{1/a} \end{array}$$

is a random variable with NE-W distribution. The random numbers are generated via the optim() R-function with the argument method = ^"^L − BFGS − B^"^. The simulation process is based on the following steps:

- Generate 750 samples of size *n* from NE-W distribution with parameters *α*, *γ*, and *θ*
- Compute the maximum likelihood estimators of (*α*, *γ*, *θ*) for *n* = 750
- Compute biases and mean square errors (MSEs) of the model parameters
- Steps (i)–(iii) are repeated for *n* = 25,50,75, ⋯, 750

**Figure 3:**
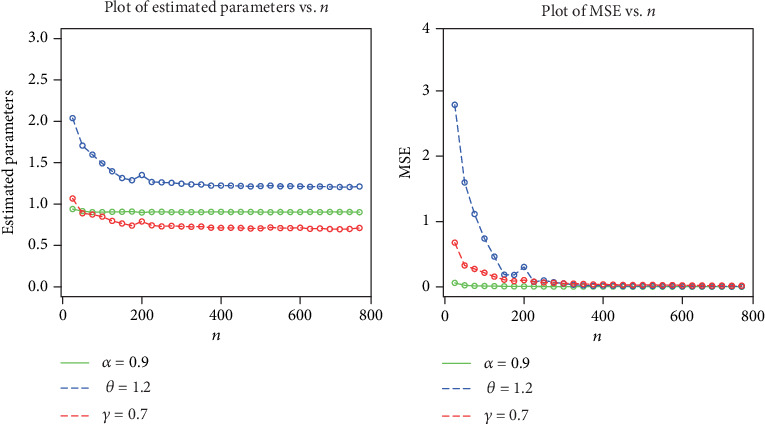
Plots of the estimated parameters and MSEs for *α* = 0.9, *θ* = 1.2, and *γ* = 0.7.

**Figure 4:**
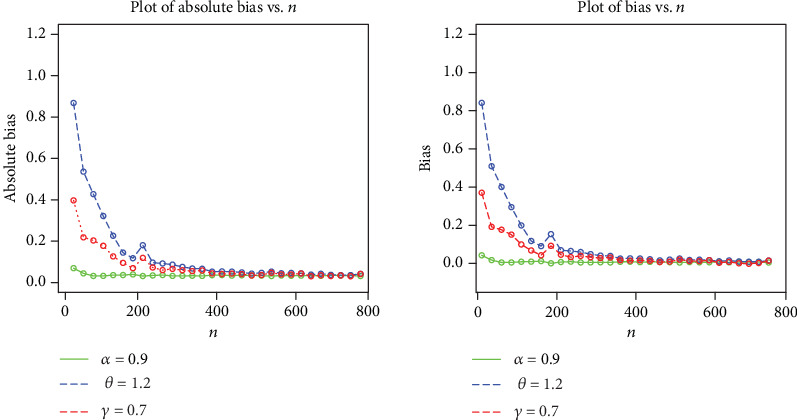
Plots of the biases and absolute biases for *α* = 0.9, *θ* = 1.2, and *γ* = 0.7.

**Figure 5:**
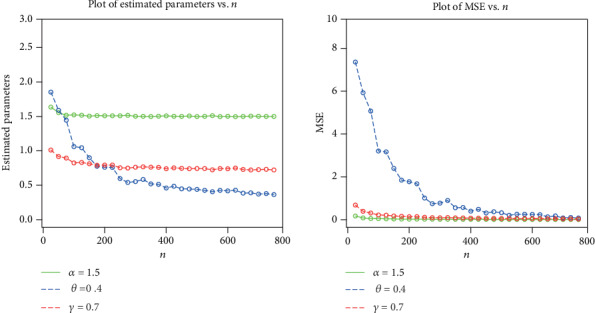
Plots of the estimated parameters and MSEs for *α* = 1.5, *θ* = 0.4, and *γ* = 0.7.

**Figure 6:**
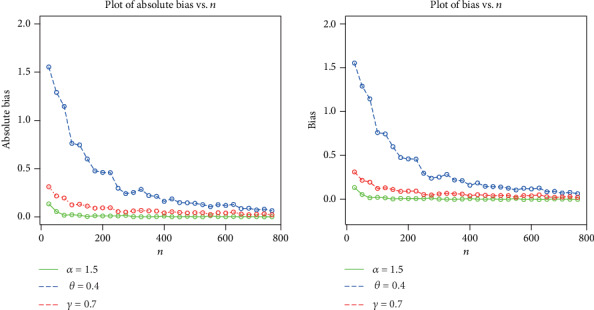
Plots of the biases and absolute biases for *α* = 1.5, *θ* = 0.4, and *γ* = 0.7.

The simulation results are provided in Figures 3–6, indicating that

- the estimates are quite stable and, more importantly, are close to the true values for these sample sizes
- the estimated biases decrease when the sample size *n* increases
- the estimated MSEs decay toward zero when the sample size *n* increases

## 5. Comparative Study

As we have mentioned earlier, the researchers have been developing new distributions to provide the best fit to real-life data in applied areas such as reliability engineering, medical, actuarial, and financial sciences. Therefore, in this section, we consider three real life applications from different discipline of applied areas including medical, engineering, and financial sciences. For each data set, the NE-W distribution is compared with different well-known distributions and we observed that the proposed distribution outclasses other competitors.

To decide about the goodness of fit among the applied distributions, we consider certain analytical measures. In this regard, we consider two discrimination measures such as the Akaike information criterion (AIC) introduced by Akaike [16] and Bayesian information criterion (BIC) of Schwarz [17], and Scollnik [18]. These following measures are given:

(i) The AIC is given by
  (21) $$\begin{array}{c} \text{AIC}=2k-2l \end{array}$$
(ii) The BIC is given by
  (22) $$\begin{array}{c} \text{BIC}=k\log\left(n\right)-2l \end{array}$$

where *ℓ* denotes the log-likelihood function evaluated at the MLEs, *k* is the number of model parameters, and *n* is the sample size. In addition to the discrimination measures, we further consider other goodness of fit measures such as the Anderson Darling (AD) test statistic, Cramer-von Mises (CM) test statistic, and Kolmogorov-Smirnov (KS) test statistic with corresponding *p* values. These following measures are given:

(i) The AD test statistic
  (23) $$\begin{array}{c} \text{AD}=-n-\frac{1}{n}\overset{n}{\underset{i=1}{\sum }}\left(2i-1\right)\left[\log G\left(x_{i}\right)+\log\left\{1-G\left(x_{n-i+1}\right)\right\}\right], \end{array}$$
 where *n* is the sample size and *x*_*i*_ is the *i*^th^ sample, calculated when the data is sorted in an ascending order
(ii) The CM test statistic
  (24) $$\begin{array}{c} \text{CM}=\frac{1}{12n}+\overset{n}{\underset{i=1}{\sum }}{\left[\frac{2i-1}{2n}-G\left(x_{i}\right)\right]}^{2} \end{array}$$
(iii) The KS test statistic is given by
  (25) $$\begin{array}{c} \text{KS}=\sup_{x}\left[G_{n}\left(x\right)-G\left(x\right)\right], \end{array}$$

where *G*_*n*_ (*x*) is the empirical cdf and sup*_x_* is the supremum of the set of distances

A distribution with lower values of these analytical measures is considered to be a good candidate model among the applied distributions for the underlying data sets. By considering these statistical tools, we observed that the NE-W distribution provides the best fit compared to other distributions because the values of all of the selected criteria of goodness of fit are significantly smaller for the proposed distribution.

### 5.1. A Real Life Application of Biomedical Analysis

The bladder cancer is the ninth most frequently diagnosed malignancy worldwide [19] and one of the most prevalent, representing 3 of cancers diagnosed globally [20]. Bladder cancer accounts for an estimated 386,000 new diagnoses and 150,000 related deaths annually. Early detection of bladder cancer remains one of the most urgent issues in many researches. The first data set is taken from Lee and Wang [21]; the authors studied the remission times (in months) of a random sample of 128 bladder cancer patients. They rejected the hypothesis of using the exponential and Weibull distributions for modeling medical sciences data having nonmonotic hazard function. The authors observed that the extended versions of these classical distributions can be used quite effectively to model such type of data. The proposed NE-W model is applied to this data in comparison with other well-known competitors. The distribution functions of the competitive models are as follows:

(1) FWE distribution
  (26) $$\begin{array}{c} G\left(x;\alpha ,\sigma ,\gamma \right)=1-\exp\left\{-e^{\sigma x^{2}-\left(\gamma /x^{\alpha }\right)}\right\},\;x\geq 0,\alpha ,\sigma ,\gamma >0 \end{array}$$
(2) APTW distribution
  (27) $$\begin{array}{c} G\left(x;\alpha_{1},\alpha ,\gamma \right)=\frac{\alpha_{1}^{\left(1-e^{-\gamma x^{\alpha }}\right)}-1}{\alpha_{1}-1},\;x\geq 0,\alpha_{1}\neq 1,\alpha ,\gamma >0 \end{array}$$
(3) Marshall-Olkin Weibull (MOW) distribution
  (28) $$\begin{array}{c} G\left(x;\alpha_{1},\alpha ,\gamma \right)=\frac{\left(1-e^{-\gamma x^{\alpha }}\right)}{\sigma +\left(1-\sigma \right)\left(1-e^{-\gamma x^{\alpha }}\right)},\;x\geq 0,\alpha ,\gamma ,\sigma >0 \end{array}$$
(4) MW distribution
  (29) $$\begin{array}{c} G\left(x;\alpha ,\gamma ,\theta \right)=1-e^{-\theta x-\gamma x^{\alpha }},\;x\geq 0,\alpha ,\gamma ,\theta >0 \end{array}$$

**Figure 7:**
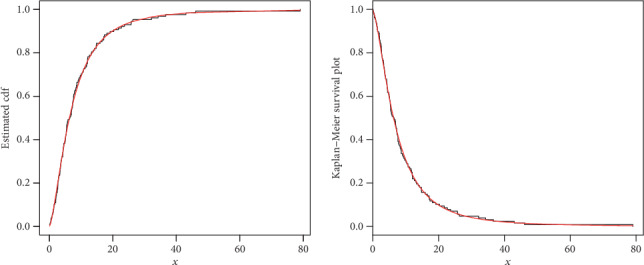
Estimated cdf and Kaplan Meier Survival plots of the NE-W distribution for data 1.

**Figure 8:**
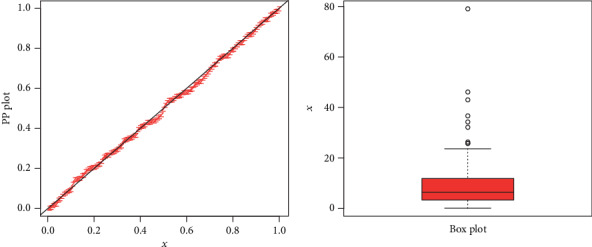
PP plot of the NE-W distribution and the box plot for data 1.

The maximum likelihood estimators with standard error (in parenthesis) of the model for the analyzed data are presented in Table 3. The discrimination measures along with the goodness of fit measures of the proposed and other competitive models are provided in Table 4. Form the results provided in Table 4, it is clear that the proposed distribution has lower values of these measures than the other models. The fitted cdf and Kaplan-Meier survival plots of the proposed model for the analyzed data set are plotted in Figure 7. The PP plot of the proposed model and Box plot of the data set are sketched in Figure 8. From Figure 7, it is clear that the proposed model fits the estimated cdf and Kaplan Meier survival plots very closely. Box plot is a tool for graphically depicting the data. It gives a good indication of how the values in the data are spread out. From Figure 8, we can easily detect that the data has a heavy tail skewed to the right (Box plot) and the proposed model closely followed the PP plot.

### 5.2. A Real Life Application from Reliability Engineering

Here, we investigate the NE-W distribution via analyzing reliability engineering data set taken from Algamal [22] representing the failure time of coating machine. To show the potentiality of the proposed method, the proposed model and other competitive distributions are applied to this data set and it is observed that the NE-W model again outclassed the well-known distributions. The distribution functions of the competitive models selected for the second data set are as follows:

(1) Ex-APTW distribution
  (30) $$\begin{array}{c} G\left(x;\alpha_{1},\alpha ,\gamma \right)=\frac{\alpha_{1}^{\left(1-e^{-\gamma x^{\alpha }}\right)}-e^{\left(1-e^{-\gamma x^{\alpha }}\right)}}{\alpha_{1}-e},\;x\geq 0,\alpha_{1},\alpha ,\gamma ,>0 \end{array}$$
(2) Ku-W distribution
  (31) $$\begin{array}{c} G\left(x,\alpha ,\gamma ,a,b\right)=1-{\left[1-{\left(1-e^{-\gamma x^{\alpha }}\right)}^{\alpha }\right]}^{b},\;x\geq 0,\alpha ,\gamma ,\alpha ,\beta >0 \end{array}$$
(3) BW distribution
  (32) $$\begin{array}{c} G\left(x,\alpha ,\gamma ,a,b\right)=I_{\left(1-e^{-\gamma x^{\alpha }}\right)}\left(a,b\right),\;x\geq 0,\alpha ,\gamma ,a,b>0 \end{array}$$

**Figure 9:**
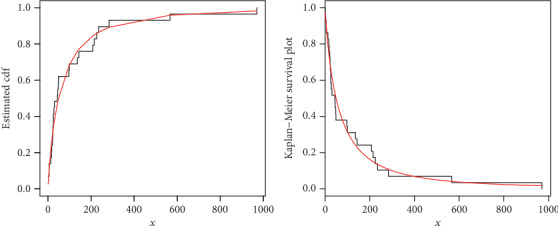
Estimated cdf and Kaplan Meier survival plots of the NE-W distribution for data 2.

**Figure 10:**
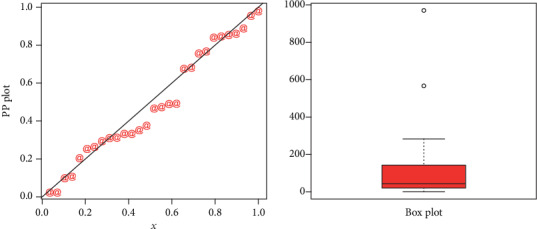
PP plot of the NE-W distribution and the box plot for data 2.

Corresponding to data set 2, the values of the model parameters are reported in Table 5. The analytical measures of the proposed and other competitive models are provided in Table 6. The estimated cdf and Kaplan-Meier survival plots are sketched in Figure 9, which shows that proposed distribution fits the estimated cdf and Kaplan-Meier survival plots very closely. The PP plot and box plot are sketched in Figure 10. From the box plot of the second data set, it is also clear that the data set has heavier tail.

### 5.3. A Real Life Application from Insurance Sciences

The third data set was taken from the insurance sciences representing the vehicle insurance losses available at http://www.businessandeconomics.mq.edu.au/our_departments/Applied_Finance_and_Actuarial_Studies/research/books/GLMsforInsuranceData. We fitted the proposed model in comparison with the other models. The distribution functions of the competitive models are as follows:

(1) Lomax
  (33) $$\begin{array}{c} G\left(x;\alpha ,\gamma \right)=1-{\left(1+\frac{x}{y}\right)}^{-\alpha },x>0,\alpha ,\gamma >0 \end{array}$$
(2) Burr
  (34) $$\begin{array}{c} G\left(x;c,k\right)=1-{\left(1+x^{c}\right)}^{-k},\;x>0,c,k>0 \end{array}$$

**Figure 11:**
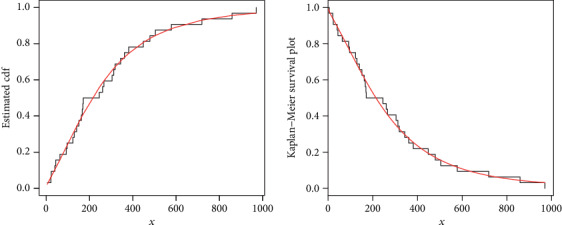
Estimated cdf and Kaplan Meier survival plots of the NE-W distribution for data 3.

**Figure 12:**
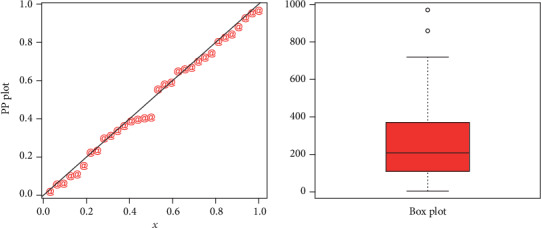
PP plot of the NE-W distribution and the Box plot for data 3.

For the third data set, parameter values are reported in Table 7, and the analytical measures are presented in Table 8. The estimated cdf and Kaplan-Meier survival plots are sketched in Figure 11. The PP plot and Box plot are sketched in Figure 12. From Figures 11 and 12, it is clear that the data set has a heavier tail and the proposed model fits the estimated cdf and Kaplan-Meier survival plots very well.

## 6. Concluding Remarks

The importance of the extended distributions was first realized in financial sciences and later in other applied fields such as engineering and medical sciences. To cater data in those fields, a number of methods have been introduced. In this context, we have proposed a versatile three-parameter distribution, called a new extended Weibull distribution using a new approach allowing closed form expressions for some basic mathematical and other related properties. The applicability of the proposed family has been illustrated via three data sets from medical, engineering, and financial sciences, and the model performs reasonably well as compared to some well-known distributions.

This new development, which has a promising approach for data modeling in the field, may be very useful for practitioners who handle such data sets. For that reason, it can be deemed as an alternative to the Weibull and other well-known competitors.

## Figures and Tables

**Table 1:** Descriptive measures of the NE-W distribution for *α* = 0.9 and *γ* = 1 and different values of *θ*.

*θ*	Mean	Variance	Sk	Kur
0.9	5.4664	130.8709	5.0976	31.7659
1.3	3.7934	51.8976	7.3847	70.2370
1.7	2.4670	20.6752	10.8760	151.0953
2.1	1.5690	9.5649	15.3218	294.3785
2.4	1.3409	4.5409	16.0965	423.0964
2.8	0.9675	2.0987	18.0967	565.9876

**Table 2:** Descriptive measures of the NE-W distribution for *θ* = 0.5 and *γ* = 1 and different values of *α*.

*α*	Mean	Variance	Sk	Kur
0.7	9.9876	311.9087	3.0987	19.9875
1.1	8.2354	236.9876	5.2398	25.7650
1.5	7.0488	155.2345	7.5467	32.0983
2.5	5.7539	45.5309	7.5680	65.3048
4.5	3.9876	30.0965	14.8654	176.8906
4.5	2.6538	15.0965	17.7856	225.1045

**Table 3:** Estimated values with standard error (in parenthesis) of the proposed and other competitive models for data 1.

Dist.	*α*	*γ*	*σ*	*α*_1_	*θ*
NE-W	1.985 (0.19654)	0.107 (0.0198)			2.156 (0.9107)
Weibull	1.047 (0.0675)	0.093 (0.0190)			
FWE	4.332 (3.5347)	0.720 (0.5492)	0.541 (0.1883)		
APTW	0.014 (0.0865)	0.016 (0.0064)		0.014 (0.0216)	
MOW	1.268 (0.1308)	0.877 (0.5205)	11.829 (11.2869)		
MW	1.007 (0.0313)	0.951 (4.2501)			0.863 (4.2551)

**Table 4:** Discrimination and goodness of fit measures of the proposed and other competitive models for data 1.

Dist.	AIC	BIC	CM	AD	KS	*p* value
NE-W	826.439	834.022	0.025	0.133	0.041	0.995
Weibull	832.173	837.877	0.131	0.786	0.069	0.558
FWE	829.219	837.775	0.051	0.329	0.049	0.910
APTW	826.378	836.934	0.042	0.255	0.045	0.949
MOW	834.988	843.544	0.150	0.884	0.075	0.451
MW	833.969	842.525	0.133	0.797	0.073	0.494

**Table 5:** Estimated values with standard error (in parenthesis) of the proposed and other competitive models for data 2.

Dist.	*α*	*γ*	*θ*	*α*_1_	*a*	*b*
NE-W	0.943 (0.3108)	2.065 (3.9765)	0.079 (0.0276)			
Ex-APTW	0.510 (0.5094)	0.172 (0.6258)		5.425 (7.0766)		
Ku-W	0.620 (0.3093)	0.501 (1.0970)			0.702 (3.2715)	0.118 (2.0964)
BW	0.478 (0.2696)	0.502 (0.5522)			2.797 (3.1595)	0.344 (0.6646)

**Table 6:** Analytical measures of the proposed and other competitive models for data 2.

Dist.	AIC	BIC	CM	AD	KS	*p* value
NE-W	332.876	335.789	0.063	0.369	0.129	0.772
Ex-APTW	335.071	339.172	0.093	0.491	0.142	0.598
Ku-W	337.750	343.220	0.091	0.546	0.146	0.488
BW	335.457	340.926	NaN	NaN	0.144	0.603

**Table 7:** The model estimators with standard error (in parenthesis) of the competing models for data 3.

Dist.	$\hat{\alpha }$	$\hat{\gamma }$	$\hat{\theta }$	$\hat{c}$	$\hat{k}$
NE-W	0.975 (0.2863)	0.031 (0.0398)	0.632 (0.3980)		
Weibull	1.019 (0.9445)	0.003 (1.6540)			
Lomax	0.495 (0.4334)	30.008 (9.6185)			
Burr				0.049 (0.1134)	4.427 (2.2671)

**Table 8:** Analytical measures of the proposed and other competing models for data 3.

Dist.	AIC	BIC	CM	AD	KS	*p* value
NE-W	427.432	432.239	0.023	0.141	0.091	0.938
Weibull	432.353	439.256	0.054	0.447	0.185	0.597
Lomax	460.191	463.021	0.083	0.520	0.207	0.108
Burr	503.477	506.383	0.228	1.362	0.416	0.208

## Data Availability

The data used to support the findings of this study are available from the corresponding author upon request.

## Appendix

Using *f*(*x*; *ξ*) = *aγx*^*a*−1^ *e*^−*γx*^*a*^^ and *F*(*x*; *ξ*) = 1 − *e*^−*γx*^*a*^^ in (18), we get the expression of the log-likelihood function for the NE-W distribution, given by

(A.1) $$\begin{array}{c} \ell_{n}\left(\Theta \right)=n\;\log\left(2\right)+2n\;\log\theta +n\;\log\alpha +n\;\log\;\gamma +\left(\alpha -1\right)\overset{n}{\underset{i=1}{\sum }}\log\;x_{i}+\overset{n}{\underset{i=1}{\sum }}\log\;A_{i}+\left(\theta -1\right)\overset{n}{\underset{i=1}{\sum }}\log\;\left(1-{\left(A_{i}\right)}^{2}\right)-\left(\theta +1\right)\overset{n}{\underset{i=1}{\sum }}\log\;\left(1-\left(1-\theta \right){\left(A_{i}\right)}^{2}\right), \end{array}$$

where *A*_*i*_ = 1 − *e*^−*γx*_*i*_^*a*^^. The partial derivatives of (A.1) are as follows:

(A.2) $$\begin{array}{c} \frac{\partial \ell_{n}\left(\Theta \right)}{\partial \theta }=\frac{2n}{\theta }+\overset{n}{\underset{i=1}{\sum }}\log\;\left(1-{\left(A_{i}\right)}^{2}\right)-\overset{n}{\underset{i=1}{\sum }}\left\{\frac{\theta {\left(A_{i}\right)}^{2}}{\left(1-\left(1-\theta \right){\left(A_{i}\right)}^{2}\right)}+\log\left(1-\left(1-\theta \right){\left(A_{i}\right)}^{2}\right)\right\}-\overset{n}{\underset{i=1}{\sum }}\frac{{\left(A_{i}\right)}^{2}}{\left(1-\left(1-\theta \right){\left(A_{i}\right)}^{2}\right)},\\ \frac{\partial \ell_{n}\left(\Theta \right)}{\partial \alpha }=\frac{n}{\alpha }+\overset{n}{\underset{i=1}{\sum }}\log x_{i}+\overset{n}{\underset{i=1}{\sum }}\left\{\frac{\left(\log x_{i}\right)x_{i}^{\alpha }e^{-\gamma x_{i}^{\alpha }}}{A_{i}}\right\}-\left(\theta -1\right)\overset{n}{\underset{i=1}{\sum }}\left\{\frac{2A_{i}\left(\log x_{i}\right)x_{i}^{\alpha }e^{-\gamma x_{i}^{\alpha }}}{1-{\left(A_{i}\right)}^{2}}\right\}+\left(\theta +1\right)\overset{n}{\underset{i=1}{\sum }}\left\{\frac{2\left(1-\theta \right)A_{i}\left(\log x_{i}\right)x_{i}^{\alpha }e^{-\gamma x_{i}^{\alpha }}}{1-\left(1-\theta \right){\left(A_{i}\right)}^{2}}\right\},\\ \frac{\partial \ell_{n}\left(\Theta \right)}{\partial \gamma }=\frac{n}{\gamma }+\overset{n}{\underset{i=1}{\sum }}\left\{\frac{x_{i}^{\alpha }e^{-\gamma x_{i}^{\alpha }}}{A_{i}}\right\}-\left(\theta -1\right)\overset{n}{\underset{i=1}{\sum }}\left\{\frac{2A_{i}x_{i}^{\alpha }e^{-\gamma x_{i}^{\alpha }}}{1-{\left(A_{i}\right)}^{2}}\right\}+\left(\theta +1\right)\overset{n}{\underset{i=1}{\sum }}\left\{\frac{2\left(1-\theta \right)A_{i}x_{i}^{\alpha }e^{-\gamma x_{i}^{\alpha }}}{1-\left(1-\theta \right){\left(A_{i}\right)}^{2}}\right\}. \end{array}$$
